# The genome anatomy of *Corynebacterium pseudotuberculosis* VD57 a highly virulent strain causing *Caseous lymphadenitis*

**DOI:** 10.1186/s40793-016-0149-7

**Published:** 2016-04-08

**Authors:** Sintia Almeida, Sandeep Tiwari, Diego Mariano, Flávia Souza, Syed Babar Jamal, Nilson Coimbra, Roberto Tadeu Raittz, Fernanda Alves Dorella, Alex Fiorine de Carvalho, Felipe Luiz Pereira, Siomar de Castro Soares, Carlos Augusto Gomes Leal, Debmalya Barh, Preetam Ghosh, Henrique Figueiredo, Lília Ferreira Moura-Costa, Ricardo Wagner Portela, Roberto Meyer, Artur Silva, Vasco Azevedo

**Affiliations:** Institute of Biologic Sciences, Federal University of Minas Gerais, Belo Horizonte, MG Brazil; Aquacen - National Reference Laboratory for Aquatic Animal Diseases, Federal University of Minas Gerais, Belo Horizonte, MG Brazil; Laboratory of Bioinformatics, Professional and Technological Education Sector, Federal University of Paraná, Curitiba, PR Brazil; Institute of Health Sciences, Federal University of Bahia, Salvador, BA Brazil; Institute of Biologic Sciences, Federal University of Para, Belem, PA Brazil; Centre for Genomics and Applied Gene Technology, Institute of Integrative Omics and Applied Biotechnology (IIOAB), Nonakuri, Purba Medinipur, West Bengal India; Department of Computer Science, Virginia Commonwealth University, Richmond, VA USA

**Keywords:** Biovar *ovis*, Gram-positive pathogen, Caseous lymphadenitis, *Corynebacterium pseudotuberculosis*, Goat, Genome sequencing, Ion Torrent PGM

## Abstract

*Corynebacterium pseudotuberculosis* strain VD57 (Cp_VD57), a highly virulent, nonmotile, non-sporulating, and a mesophilic bacterium, was isolated from a goat’s granulomatous lesion in the municipality of Juazeiro, Bahia State, Brazil. Here, we describe a set of features of the strain, together with the details of its complete genome sequence and annotation. The genome comprises of a 2.5 Mbp long, single circular genome with 2,101 protein-coding genes, 12 rRNA, 49 tRNA and 47 pseudogenes and a G + C content of 52.85 %. Genetic variation was detected in Cp_VD57 using *C. pseudotuberculosis* strain 1002 as reference, wherein small genomic insertions and deletions were identified. The comparative analysis of the genome sequence provides means to better understand the host pathogen interactions of this strain and can also help us to understand the molecular and genetic basis of virulence of this bacterium.

## Introduction

*Corynebacterium pseudotuberculosis* is the etiologic agent of caseous lymphadenitis in sheep and goats, the organism has also been associated with mastitis [1–3] and can cause ulcerative lymphangitis in horses and cattle [4]. CL is a chronic disease that is characterized by the formation of granulomas in lymph nodes and internal organs, as a response of the host’s immune system against this bacterium that resists to the bactericidal action of phagocytic cells [3].

CL is considered as one of the economically important diseases of small ruminants with losses attributed to reduced wool and hide yields, carcass condemnation, morbidity and rarely mortality [5, 6]. The prevalence of CL has been observed worldwide, including South Africa, Brazil, the USA, Canada, Australia, New Zealand, United Kingdom and Egypt [7].

The pangenome analysis of 15 strains of the pathogen was completed recently [8]. However, as *C. pseudotuberculosis* is a relatively clonal organism [9–13], the identification of the virulence mechanisms or nucleotide modifications responsible for making a strain more virulent than another, have not yet been identified.

Sequencing of new genomes coupled with a deeper comparative analysis between the genomes and associating such analyses with the host pathogen interactions can help us understand and identify the differences between genomes and virulence factors. In this context, the present study reports the sequence the genome of the highly virulent strain VD 57 and to understand its virulence factors.

## Organism information

### Classification and features

*C. pseudotuberculosis* is a Gram-positive bacteria and belong to a CMNR *(**Corynebacterium**,**Mycobacterium**,**Nocardia* and *Rhodococcus*) group that shares characteristics including an outer lipid layer, mycolic acids in the cell wall along with its derivatives including phospholipids and lipomannans [7]. *C. pseudotuberculosis* is a facultative intracellular pathogen showing pleomorphic forms like coccoids and filamentous rods, non-motile, non-sporulating and possessing fimbriae, with sizes ranging between 0.5–0.6 μm and 1.0–3.0 μm [7].

The *C. pseudotuberculosis* strain VD57 (Cp_VD57) was isolated from a goat’s granulomatous lesion in the municipality of Juazeiro, Bahia State, Brazil. The bacterial identification was made through Gram’s staining, colonies’ morphology analysis, synergic hemolysis with *Rhodococcus equi* in Brain Heart Infusion, Blood Agar Medium, and biochemical assays using the API Coryne system (BioMérieux). The strain is maintained in BHI broth at the Microbiology Laboratory of the Federal University of Bahia [14, 15].

*C. pseudotuberculosis* strain VD57 has been shown to be highly pathogenic to goats and mice [14]. This Cp_VD57 strain was able to induce IFN-gamma production in goats on day 5 after infection. Additionally, it induced a positive antibody titer between 6 and 11 days after infection [16]. Using a murine experimental model, it was observed that, the strain was able to induce a high mortality, when compared to the T1 attenuated strain, confirming its virulent profile [15]. Moura-Costa et al. used Cp_VD57 strain to challenge goats that were immunized with the attenuated T1 strain, obtaining a protection of 33.3 % and a strong humoral response, but the immunization was not able to prevent the spread of this virulent bacteria in the majority of the vaccinated animals [14].

**Fig. 1:**
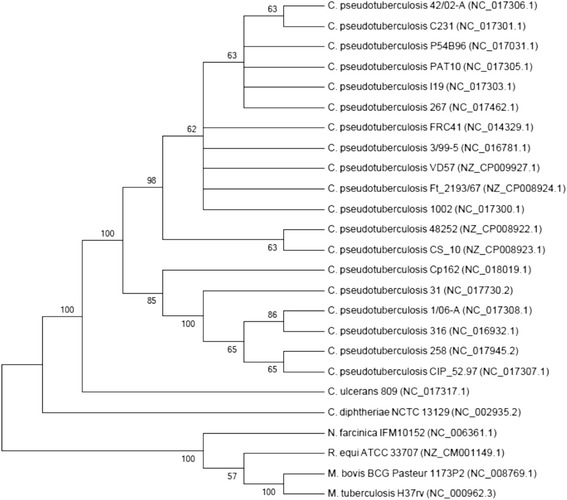
Phylogenetic tree of *C. pseudotuberculosis* strain VD57 representing its position relative to type strains in *Corynebacteriaceae* along with some other type strains of CMNR group. The tree was inferred from 3,537 aligned characters of the *rpoB* gene sequence using maximum likelihood method and then checked for its agreement with the current classification in Table 1. The branch lengths represent the expected number of substitutions per site. Numbers adjacent to the branches are support values from 1,000 bootstrap replicates, indicated when larger than 60 %. Calculations to determine the phylogenetic distances were done by the software MEGA v6 [40]. The GenBank accession numbers are shown in parentheses

One of the most important fields in the *C. pseudotuberculosis* study is the definition of genes that are differentially expressed in bacterial cultures and inside the granulomatous lesions. In this regard, VD57 strain was used in a study with the objective to determine reference genes to be used in quantitative real time PCR. It was found that eight of these genes (*atpA*, *dnaG*, *efp*, *fusA*, *gyrA*, *gyrB*, *rpoB*, and *rpoC*), mostly participating in DNA replication and transcription, can be useful as candidate reference genes, while DNA gyrase subunit A (*gyrA*) and elongation factor P (*fusA*) presented the most suitable profiles to be used in qPCR studies [17]. Figure 1 shows a phylogenetic tree of *Corynebacterium pseudotuberculosis* strain VD57 based on *rpoB* gene (β subunit of RNA polymerase). All the classification and general features of *C. pseudotuberculosis* strain VD57 are summarized in Table 1.

**Table 1:** Classification and general features of *Corynebacterium pseudotuberculosis* strain VD57 according to the MIGS recommendations [19]

MIGS ID	Property	Term	Evidence code^a^
	Classification	Domain *Bacteria*	TAS [30]
		Phylum *Actinobacteria*	TAS [31]
		Class *Actinobacteria*	TAS [32]
		Order *Actinomycetales Suborder Corynebacterineae*	TAS [32, 33]
		Family *Corynebacteriaceae*	TAS [32–35]
		Genus *Corynebacterium*	TAS [36–38]
		Species *Corynebacterium pseudotuberculosis*	TAS [37, 39]
	Gram stain	Positive	TAS [14]
	Cell shape	*Bacilli*	TAS [14]
	Motility	Non-motile	TAS [14]
	Sporulation	Non-sporulating	TAS [14]
	Temperature range	Mesophilic	NAS
	Optimum temperature	37 °C	TAS [14, 18]
	pH range; Optimum	7.0–7.2	TAS [7]
	Carbon source	Glucose	TAS [14]
MIGS-6	Habitat	Host	TAS [32]
MIGS-6.3	Salinity	Not reported	
MIGS-22	Oxygen requirement	Aerobic and Obligate Aerobic	TAS [14, 18]
MIGS-15	Biotic relationship	Intracellular facultative pathogen	TAS [7, 14, 15]
MIGS-14	Pathogenicity	Goat	TAS [14]
MIGS-4	Geographic location	Bahia State, Brazil	TAS [14]
MIGS-5	Sample collection time	2005	[NAS]
MIGS-4.1	Latitude	9°24’S	[IDA]
MIGS-4.2	Longitude	40°30’W	[IDA]

^a^Evidence codes - *IDA* Inferred from Direct Assay, *TAS* Traceable Author Statement (i.e., a direct report exists in the literature), *NAS* Non-traceable Author Statement (i.e., not directly observed for the living, isolated sample, but based on a generally accepted property for the species, or anecdotal evidence)

De Souza et al. employed VD57 strain to verify the intracellular signaling cascade activation during the infection of splenocytes with the bacterium, and the importance of signaling pathways in the production of different cytokines. The results showed that VD57 strain was able to induce the production of TNF-alpha through the MAPK p38, and IL-10 induction via ERK-1 and −2 pathways. The complete genome sequencing and analysis will help in identifying the genetic background and the genes that may be involved in the infections [18].

## Genome sequencing information

### Genome project history

In the present study, we determined the nucleotide sequence of the *C. pseudotuberculosis* strain VD57 (Cp_VD57) genome, isolated from a goat granulomatous lesion. Sequencing, assembly, and annotation were performed at Laboratory of Cellular and Molecular Genetics (LGCM), Federal University of Minas Gerais, Belo Horizonte, Minas Gerais, Brazil and Aquacen - National Reference Laboratory for Aquatic Animal Diseases, Federal University of Minas Gerais, Brazil. The Cp_VD57 complete genome sequence and annotation data were deposited in the GenBank under the accession number CP009927. Table 2 presents the project information in accordance with the Minimum Information about a Genome Sequence (MIGS) [19].

**Table 2:** Genome sequencing project information

MIGS ID	Property	Term
MIGS 31	Finishing quality	Finished
MIGS-28	Libraries used	Fragments
MIGS 29	Sequencing platforms	Semiconductor Ion Torrent PGM
MIGS 31.2	Fold coverage	78.22-fold
MIGS 30	Assemblers	MIRA .4.0CLC Genome Workbench 4.7.2
MIGS 32	Gene calling method	Glimmer v3.02
	Locus Tag	CpVD57
	Genbank ID	CP009927 (chromosome)
	GenBank Date of Release	January 06, 2015
	BIOPROJECT	PRJNA267107
MIGS 13	Source Material Identifier	BHI broth, VD57
	Project relevance	Animal Pathogen, Medical

### Growth conditions and genomic DNA preparation

Cp_VD57 strain was grown in brain-heart-infusion media (BHI-HiMedia Laboratories Pvt. Ltd, India) under rotation at room temperature (37 °C). Extraction of chromosomal DNA was performed using 30 mL of 48–72 h culture of bacteria, centrifuged at 4 °C and 4000 rpm for 15 min. Re-suspension of cell pellets was done in 600 μL Tris/EDTA/NaCl [10 mM Tris/HCl (pH7.0), 10 mM EDTA (pH 8.0), and 300 mM NaCl], and transferred to tubes with beads for cell lysis using Precellys®24-Dual (2 cycles of 15 s at 6500 rpm with 30 s between them). Thereafter, purification of DNA with phenol/chloroform/isoamyl alcohol (25:24:1) was followed by precipitation with ethanol/NaCl/glycogen (2.5v, 10 % NaCl and 1 % glycogen). The DNA was re-suspended in 30 μL MilliQ®. The concentration was determined by spectrophotometer, and the DNA was visualized in ethidium bromide-stained 0.7 % agarose gel.

### Genome sequencing and assembly

The Ion Personal Genome Machine® System (Life Technologies) platform was used for sequencing, using fragment library. The reads with good quality was assembled using *de novo* strategy through Mira 4.0 software [20]. The assembly produced a total of 15 contigs, coverage of 78.22x with a N_50_ contig length of 405.436. Additionally, a scaffold was created using the CONTIGuator 2 software [21], taking the genome sequence of *C. pseudotuberculosis* strain 1002 (NC_017300.1) as reference. The gaps were closed manually using CLC Genomics Workbench 7 software [22].

### Genome annotation

The annotation of genes was transferred by our in-house scripts using *C. pseudotuberculosis* strains 1002, 258 (NC_017945.2) and FRC41 (NC_014329.1) as reference. Manual annotation was performed using Artemis software [23]. Other elements such as rRNA, tRNA, and repetitive regions were predicted using RNAmmer [24], tRNAscan-SE [25], and Tandem Repeat Finder [26], respectively. Enzyme Commission Numbers (EC number) prediction were performed using RAST tool [27].

## Genome properties

**Fig. 2:**
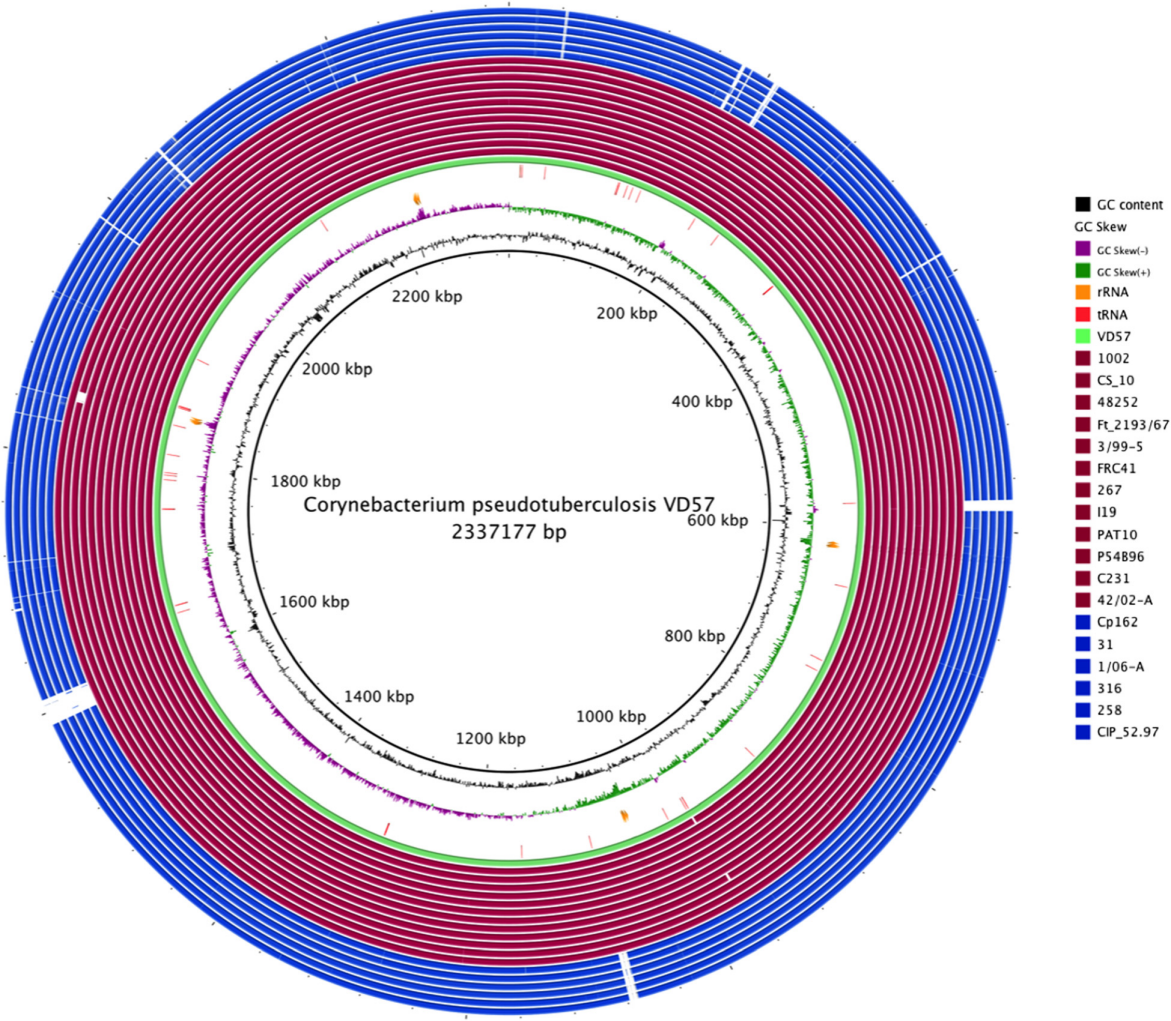
Graphical circular map of the genome [41]. From center to the outside: In wine Ovis strains, in blue Equi strains, RNA genes (tRNAs green, rRNAs orange, tRNAs red), GC content in black, GC skew

The genome is 2,337,177 bp long and comprises one main circular chromosome with a 52.19 % GC content. A total of 2,148 genes were predicted, among which 2,101 were protein coding genes, and 61 RNAs. Forty seven pseudogenes were also identified. The properties and statistics of the Cp_VD57 strain genome are listed in Table 3. The distributions of genes according to the COGs functional categories is presented in Table 4, followed by a cellular overview diagram in Fig. 2 and a summary of metabolic network statistics shown in Table 5.

**Table 3:** Genome Statistics

Attribute	Value	% of Total
Genome size (bp)	2,337,177	100.0
DNA coding (bp)	1,998,286.	85.5
DNA G + C (bp)	1,235,198	52.9
DNA scaffolds	1	
Total genes^a^	2,148	100.0
Protein coding genes^a^	2,101	97.8
RNA genes	61	2.83
Pseudo genes	47	2.2
Genes in internal clusters	NA	NA
Genes with function prediction	1,578	73.5
Genes assigned to COGs	1,629	75.8
Genes with Pfam domains	1,682	80,1
Genes with signal peptides	158	7.36
Genes with transmembrane helices	605	28.8
CRISPR repeats	NA	NA

^a^The total is based on either the size of the genome in base pairs or the total number of protein coding genes in the annotated genome

**Table 4:** Number of genes associated with the general COG functional categories

Code	Value	% age^a^	Description
J	148	7.04	Translation, ribosomal structure and biogenesis
A	2	0.09	RNA processing and modification
K	113	5.37	Transcription
L	104	4.95	Replication, recombination and repair
B	0	0.00	Chromatin structure and dynamics
D	20	0.95	Cell cycle control, cell division, chromosome partitioning
Y	0	0.00	Nuclear structure
V	31	1.47	Defense mechanisms
T	51	2.42	Signal transduction mechanisms
M	93	4.42	Cell wall/membrane biogenesis
N	5	0.23	Cell motility
Z	1	0.04	Cytoskeleton
W	0	0.0	Extracellular structures
U	33	1.57	Intracellular trafficking and secretion
O	82	3.90	Posttranslational modification, protein turnover, chaperones
C	100	4.75	Energy production and conversion
G	115	5.47	Carbohydrate transport and metabolism
E	191	9.09	Amino acid transport and metabolism
F	69	3.28	Nucleotide transport and metabolism
H	103	4.90	Coenzyme transport and metabolism
I	62	2.95	Lipid transport and metabolism
P	128	6.09	Inorganic ion transport and metabolism
Q	31	1.47	Secondary metabolites biosynthesis, transport and catabolism
R	193	9.18	General function prediction only
S	141	6.71	Function unknown
-	472	22.46	Not in COGs
Total^b^	2288	104.42	

^a^The percentage is based on the total number of protein coding genes in the annotated genome

^b^The total does not correspond to 1,537 CDSs, because some genes are associated with more than one COG functional categories

**Table 5:** Metabolic Network Statistics

Attribute	Value
Total genes	2145
Enzymes	599
Enzymatic reactions	1197
Metabolic pathways	232
Compounds	912

## Insights from the genome sequence

Genetic variation seems to be limited in *C. pseudotuberculosis*, which has been shown previously as genetically homogenous [9–13]. The MLST findings of the 64 biovar ovis strains show seven STs and all were clonally derived by eBURST analysis when a complex was deemed to share 7/8 loci; the strain Cp_VD57 was included in this analysis [28]. Although it is evident that there is very little genetic variation, we analyzed the fully sequenced Cp_VD57 genome to detect the presence of SNPs. The detected SNPs are listed in Table 6.

**Table 6:** Total number of SNP’s in *C. pseudotuberculosis* VD57 in comparison to other strains

Reference	Total SNPs	SNP coding regions	SNP intergenic regions
*C. pseudotuberculosis* 31 Equi	25,609	19,811	5,798
*C. pseudotuberculosis* 258 Equi	25,706	21,303	4,403
*C. pseudotuberculosis* 106A Equi	24,352	18,085	6,267
*C. pseudotuberculosis* 5297 Equi	25,866	20,017	5,849
*C. pseudotuberculosis* 162 Equi	24,274	18,501	5,773
*C. pseudotuberculosis* 316 Equi	25,905	20,911	4,994
*C. pseudotuberculosis* 1002 Ovis	35	28	7
*C. pseudotuberculosis* C231 Ovis	952	741	211
*C. pseudotuberculosis* P54B56 Ovis	999	754	245
*C. pseudotuberculosis* I19 Ovis	968	762	206
*C. pseudotuberculosis* FRC41 Ovis	471	374	97
*C. pseudotuberculosis* 267 Ovis	2,404	1,869	535
*C. pseudotuberculosis* PAT10 Ovis	1,060	804	256
*C. pseudotuberculosis* 4202 Ovis	956	735	221
*C. pseudotuberculosis* 3/99-5 Ovis	502	411	91
*C. pseudotuberculosis* 48252	521	394	127
*C. pseudotuberculosis* CS_10	516	392	124
*C. pseudotuberculosis* Ft_2193	492	380	112

To run SNP detection programs with MUMmer [29], default parameters were assigned. The results for SNP are in agreement with the literature, despite the fact that these strains were isolated from several hosts in different countries thereby verifying that *C. pseudotuberculosis* strains show limited genetic differences between worldwide strains.

Small genomic insertions and deletions were identified using the reference strain 1002, which is closer to Cp_VD57. MUMmer [29] identified 425 indels in Cp_VD57, 18 of which were in coding regions. However, three major regions of indel were identified comparing 1002 and VD57 strains: two insertion regions and one deletion. The first insertion region is located at coordinates 966430 to 968875 and comprises 2445 pb; this region has 4 genes and is present in biovar Equi strains. The second insertion region is located at coordinates 1182765 to 1182855 (90 pb), and is located within a hypothetical protein. Finally, the deletion region is located at 1002 strain (1575360–1576000) and comprises 640pb *aceF* pseudogenes.

## Conclusions

Isolates from the *C. pseudotuberculosis* are genetically homogenous. Multi-locus sequence typing and comparative genomic analysis show that the isolates ovis seem to fall into the same clades. Despite the general similarity between the strains from *C. pseudotuberculosis*, some are more virulent, as *C. pseudotuberculosis* strain VD57 presented in this paper. Comparative studies with genome sequences of different *C. pseudotuberculosis* strains and Cp_VD57 can be performed and these analyses may be useful in identification of genome variations.

## Abbreviations

CL: caseous lymphadenitis
Cp_VD57: *Corynebacterium pseudotuberculosis* strain VD57
